# *Moringa oleifera* Seed Cake as a Promising Prototype for Designing Phyto-Protectants Against *Fusarium oxysporum* f. sp. *lycopersici* in Tomato

**DOI:** 10.3390/ijms27135788

**Published:** 2026-06-26

**Authors:** Gina Rosalinda De Nicola, Cono Vincenzo, Catello Pane

**Affiliations:** 1 Consiglio per la Ricerca in Agricoltura e l’Analisi dell’Economia Agraria (CREA), Centro di Ricerca Orticoltura e Florovivaismo, Via dei Fiori 8, 51017 Pescia, Italy; 2 Consiglio per la Ricerca in Agricoltura e l’Analisi dell’Economia Agraria (CREA), Centro di Ricerca Orticoltura e Florovivaismo Via Cavalleggeri 51, 84098 Pontecagnano Faiano, Italy; cono.vincenzo@crea.gov.it (C.V.); catello.pane@crea.gov.it (C.P.); 3 Department of Agricultural Sciences, University of Naples Federico II, 80055 Portici, Italy

**Keywords:** *Moringa oleifera*, glucomoringin, moringin, *Fusarium oxysporum*, biopesticide, valorization, byproduct, antioxidant activity, sustainability, circular economy

## Abstract

*Moringa oleifera* seed cake is the byproduct of moringa oil extraction and the most valuable source of 4-(α-L-rhamnosyloxy)benzyl glucosinolate (glucomoringin; GMG), the precursor of 4-(α-L-rhamnosyloxy)benzyl isothiocyanate (moringin; GMG+M). The vascular fungus *Fusarium oxysporum* f. sp. *lycopersici* (FOL) is an important soil-borne pathogen of tomato in cultivated areas worldwide. Coating seeds with phytochemicals has been reported to prevent seed transmission and control seedling infection. In this work, GMG was extracted and purified from moringa seed cake on the multigram scale, and GMG+M solutions obtained through controlled hydrolysis of the precursor with commercial myrosinase were evaluated against the pathogen both in vitro and in planta. FOL conidia germination and mycelial growth were significantly inhibited by GMG+M solutions in the range 1–1000 µM, in a dose-dependent manner, compared to GMG and control treatments, which did not differ significantly. Interestingly, the coating of tomato var. crovarese seeds with GMG or GMG+M (100 µM) resulted in equally effective reduction (70%) of the disease severity in post-emergence, suggesting a plant-mediated mechanism underlying the efficacy of the intact glucosinolate. Seed coating with both phytochemicals triggered polyphenol oxidase (PPO) activity in five-day-old tomato sprouted rootlets. This study highlighted the potential biotechnological value of *M. oleifera* seedcake for the development of a sustainable biopesticide.

## 1. Introduction

*Moringa oleifera* Lam. (Moringaceae) is a species native to the diversification centers of South Asia, also known as the horseradish or drumstick tree, or by other common names including the miracle tree in different tropical and subtropical regions where it is cultivated and used for wood, food, and industrial applications [1]. This multipurpose crop is exploited for oil extraction from its seeds (about 6–7000 seeds per tree, equal to 45–48 kg of biomass) with valuable qualitative and quantitative composition in fatty acids, and for its good yield, estimated at 900 kg of oil per hectare [2,3,4]. The main byproduct of the oil extraction process is the moringa seed cake, rich in protein and carbohydrates, suitable for a range of applications including food production [5], vegetal protein sourcing [6], and the extraction of bioactive phytochemicals [7]. On the one hand, *M. oleifera* seed has been extensively explored for its phytochemical properties, which have proven health-promoting potential, exerting a broad biological functionality [8], including antimicrobial [9], anti-inflammatory [10], antioxidant [11] and antiproliferative [12] activity. On the other hand, moringa seed cake represents a valuable source for obtaining crude or partially purified extracts (i.e., organic solvent- or water-based) [13,14], proteins (i.e., chitin-binding protein) [15], as well as purified molecules (i.e., peptides, isothiocyanates) [16,17] with direct biocidal action against fungal pathogens. These studies paved the way to further investigate moringa seed cake as a source of alternatives for the sustainable protection of agricultural crops [18].

**Figure 1:**
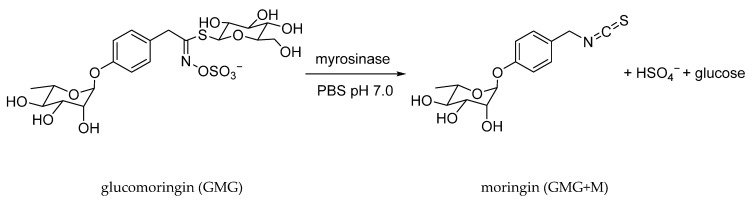
Reaction of myrosinase (β-thioglucoside glucohydrolase; EC 3.2.1.147) catalyzed hydrolysis of 4-(α-L-rhamnosyloxy)benzyl glucosinolate (glucomoringin; GMG) in phosphate-buffered solution (pH 7.0) to produce 4-(α-L-rhamnosyloxy)benzyl isothiocyanate (moringin; GMG+M).

Moringa is the sole genus in the family Moringaceae, consisting of 13 different species [19], and as a member of the order Brassicales, it is distinguished by the presence of the so-called ‘mustard oil bomb’, the glucosinolate–myrosinase system, which is the endogenous defense setup against pests and diseases [20]. Notably, the chemical tag of *M. oleifera* is 4-(α-L-rhamnopyranosyloxy)benzyl glucosinolate (commonly named glucomoringin; GMG), a structurally unusual *O*-glycosylated form of 4-hydroxybenzyl glucosinolate (glucosinalbin; SNB), which is instead widespread in several families of the order Brassicales. Particularly, moringa seeds accumulate GMG up to 8–10% of their dry weight, which offers considerable valuable potential to the coproduct of oil extraction [21]. Indeed, GMG can be extracted from moringa seed cake and transformed into the corresponding 4-(α-L-rhamnopyranosyloxy)benzyl isothiocyanate (commonly named moringin; GMG+M) by myrosinase (β-thioglucoside glucohydrolase; EC 3.2.1.147) catalyzed hydrolysis at neutral pH (Figure 1) [22,23].

Agricultural crops, such as tomatoes, need protection against biotic stress factors, such as fungal pathogens that cause harmful diseases that negatively affect yields and result in economic losses for farmers. In a regulatory and market framework geared towards reducing the use of synthetic pesticides in agriculture, there is growing interest in researching nature-based alternatives for sustainable, optimized, and residue-free crop protection. Tomato is a widespread crop all over the world, cultivated in greenhouses and in open fields, both for fresh and canned consumption. In 2024, the global production was estimated at about 188.5 million tons on a harvested area of around 5.1 million ha, with an average yield of 36.8 Mg ha^−1^ [24]. Among the limiting factors that affect their agronomic performance, the susceptibility of tomato plants to many telluric pathogens is highly impactful, and it needs to be managed. In addition to leading to high risks of fruit loss, their preventive and curative control relies on the use of synthetic chemical fungicides, raising issues of environmental externality. *Fusarium oxysporum* f. sp. *lycopersici* (Sacc.) Snyder and H.N.W. (FOL) is a very feared fungal soil-borne pathogen of tomato, causing wilting, which can cause significant reductions in crop productivity [25]. The pathogen has multiple propagation possibilities that make it insidious, through infected tissues and seeds contaminated with mycelium and/or conidia, as well as through conservative structures (chlamydospores) that reside in the soil [26]. Under favorable pedoclimatic conditions, it enters the host root and moves inside, colonizing first the cortical tissues and then the vascular ones, resulting in a gradual reduction until complete loss of the lymph flow function that normally ensures the absorption of water and nutrients. Diseased plants show a progression of symptoms passing from yellowing and stunting growth to withering and wilting, as the damage at the xylem levels in the root system and collar increases [27].

Control strategies range from the systematic use of synthetic fungicides to genetic resistance induction. Available options cover physical methods, applied agroecology, and biological control, including microorganisms and phytochemicals, with targeted soil management and/or direct treatment of parts of the canopy and/or preventive treatment of seeds [26,28]. The available literature on the topic provides a broad overview of plant extracts tested for their potential effectiveness against Fusarium wilt in tomatoes [29], with evidence of mechanisms of action that can be either direct against the pathogen [30] or plant-mediated through enhanced plant immunity and physiological response [31]. This framework provided the basis for research aimed at developing plant fungicides with specific application design [32]. Tomato Fusarium wilting has been the target, for example, of some interesting studies focused on the development of biofungicides based on extracts from glucosinolate-containing Brassicaceae vegetable matrices, such as root, leaf and fruit of radish [33]. However, despite the proven anti-soil-borne pathogen biofumigant capacity of Brassicaceae biomass [34], the application of glucosinolate-based extracts to specific crops for the control of this specific disease is still rarely used. It is therefore necessary to expand the research into the biofungicidal properties of this promising class of phytochemicals, also with a view to developing targeted application methods.

**Figure 2:**
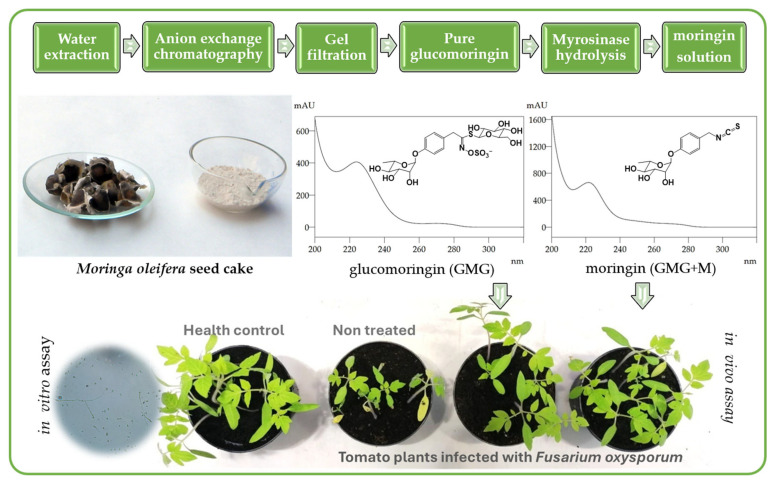
Summary of the experimental workflow carried out in this study. *Moringa oleifera* seed cake was efficiently used to obtain pure glucomoringin (GMG) on the multigram scale through a two-step chromatographic process. Pure GMG was then biotransformed with myrosinase to the isothiocyanate moringin (GMG+M). The potential of both phytochemicals to control the pathogen *Fusarium oxysporum* f. sp. *lycopersici* (FOL) was evaluated in in vitro and in vivo disease assays.

The aim of this research, as summarized in Figure 2, was to assess the antifungal activity of pure GMG obtained from *M. oleifera* seed cake on the multigram-scale level, and its myrosinase-mediated hydrolysis isothiocyanate GMG+M, on the conidia germination and mycelial growth of FOL. In addition, in vivo disease assays were used to assess the efficacy of seed coating treatments with both phytochemicals in reducing vascular wilt in tomato.

## 2. Results

### 2.1. Glucomoringin Purification

GMG was purified on the multigram scale through a two-step chromatographic process, as already reported in numerous research studies requiring a large amount of the pure compound for animal model studies [35,36,37]. HPLC-PDA analysis revealed GMG (peak of desulfo-GMG eluted at retention time (rt) 13.3 min) as a single glucosinolate present in moringa seed cake PKM-2 with a remarkable content of 13.9% (*w*/*w*). Boiling water enabled the extraction of GMG with a process efficiency of 80%. The freeze-dried water extract resulted in a white powder highly enriched in GMG, containing 32.0% (*w*/*w*) of the target glucosinolate. Afterwards, GMG was first isolated by anion exchange chromatography and then further purified by gel filtration. The entire workflow applied to 100 g of moringa seed cake afforded 6.1 g of purified GMG as a fine white powder after freeze-drying. The purity of GMG assessed by HPLC-PDA was 99% (area peak based) and 95% on a weight basis, due to its high hygroscopicity. Overall, pure GMG was obtained with a total yield of 43.9% based on the starting raw material.

### 2.2. Glucomoringin Biotransformation into Moringin

Purified GMG was biotransformed into the corresponding isothiocyanate GMG+M after preincubation with myrosinase enzyme. The biotransformation was controlled by HPLC-PDA analysis monitoring the reduction in GMG peak (rt 6.6 min) until its complete disappearance in the reaction mixture, yielding GMG+M (peak eluted at rt 41.1 min) quantitatively. This strategy of obtaining fresh solutions of GMG+M has already been extensively reported in research requiring the administration of bioactive GMG+M to mice or rats in animal model studies [35,36,37], as well as in a study addressing the antibacterial potential of GMG+M against nosocomial pathogens [22].

### 2.3. In Vitro Antifungal Activity

**Figure 3:**
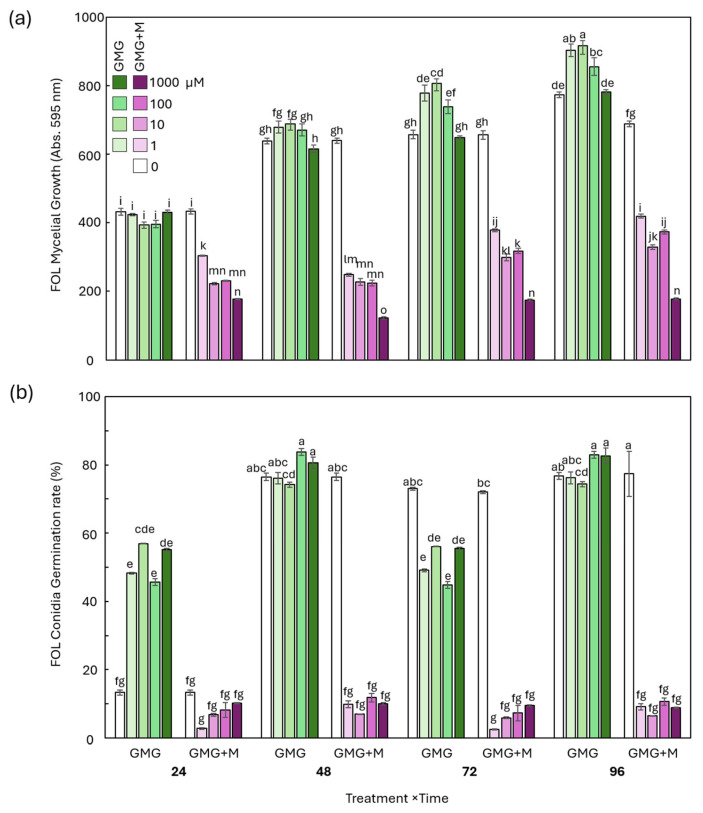
Effect of glucomoringin (GMG) and glucomoringin hydrolyzed by myrosinase (GMG+M) solutions, applied at concentrations in the range 0–1000 µM, on *Fusarium oxysporum* f. sp. *lycopersici* (FOL) mycelial growth expressed as OD value at 595 nm (**a**) and conidia germination rate percentage (**b**). Time is expressed as hours post-inoculum. Bars are the mean values ± standard error; different lowercase letters indicate significant differences among bars according to the Tukey HSD test (*p* ≤ 0.05).

Treatments with the isothiocyanate GMG+M incited significant inhibitory effects over time on both FOL mycelial growth (Figure 3a) and FOL conidia germination rate in liquid microcultures (Figure 3b). As a matter of fact, Table 1 reports synthetic results of statistics applied to in vitro assays. The fungus propagules reached the maximum germination rate in the untreated control after 48 h post-inoculum, whereas treatments with GMG showed no significant suppressive effects. At the same post-inoculum time, conversely, exposure of FOL conidia to GMG+M caused a marked reduction in the range 60–80% of mycelial growth measured in terms of absorbance at 595 nm and an 85–90% decrease in germination rate. Mycelial growth assessed spectrophotometrically was shown to be inhibited by GMG+M in a dose-dependent manner, being completely arrested by the highest concentration tested (Figure 3a). Very low levels of germination were, instead, observed at all tested concentrations of the freshly prepared GMG+M solution (Figure 3b).

### 2.4. In Vivo Disease Control Ability on Tomato Plants

**Figure 4:**
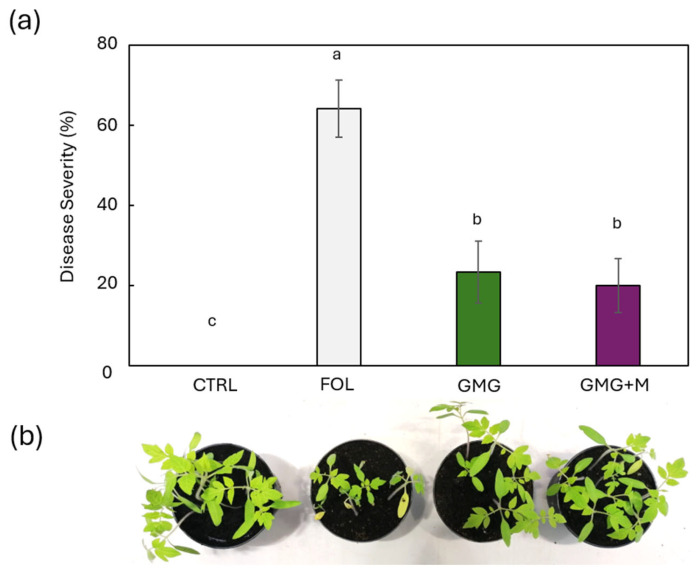
(**a**) Effect of glucomoringin (GMG) and glucomoringin hydrolyzed by myrosinase (GMG+M) solutions, used for seed coating at a concentration of 100 µM, on disease severity percentage of tomato wilting caused by *Fusarium oxysporum* f. sp. *lycopersici* (FOL) in comparison to untreated inoculated (FOL) and non-inoculated (CTRL) control plants. Bars are the mean values ± standard error; different lowercase letters indicate significant differences between bars according to the Tukey HSD test (*p* ≤ 0.05). (**b**) Photograph of representative pots per treatment.

Seed treatments with both pure GMG and GMG+M solutions increased the fitness of the seedlings grown under the artificial infection of the substrate with FOL. Wilting incidence stood, on average, at 47% of sown seeds in the untreated control, while it reached a maximum of 25% of sown coated seeds (Figure 4b). Similarly, disease severity on plants grown from GMG and GMG+M-coated seeds was reduced, on average, by 64% and 68%, respectively, compared to the FOL-infected control, which showed about 65% severity assessed at the end of the 21-day pot trial (Figure 4a). However, plants grown from seeds coated with the two phytochemical treatments did not show statistically significant differences in disease severity. Particularly in the bio-treated pots, most of the pathogen damage occurred at germination, with losses due to damping-off. In the FOL-infected control, even plants that managed to escape the initial risks of damping-off showed stunted growth and widespread yellowing, which are the typical symptoms of the Fusarium wilt.

### 2.5. Effect of Seed Coating on Oxidative Stress in Tomato Sprouted Rootlets

**Figure 5:**
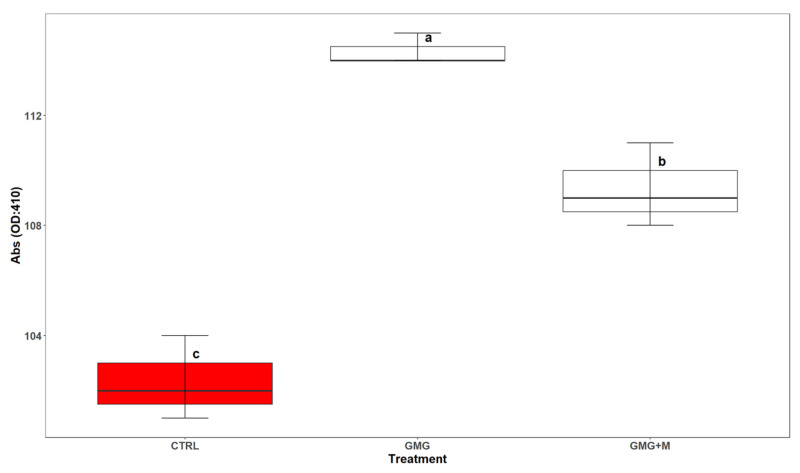
Effect of glucomoringin (GMG) and glucomoringin hydrolyzed by myrosinase (GMG+M) solutions on polyphenol oxidase (PPO) determined in sprouted rootlets developed from seeds treated with 100 µM phytochemical solutions, compared to untreated control (CTRL). Bars are the mean values ± standard error; different lowercase letters indicate significant differences among bars according to the Tukey HSD test (*p* ≤ 0.05).

Sprouted rootlets developed from tomato seeds treated with 100 µM GMG and GMG+M solutions showed higher levels of PPO activity compared to the non-treated control. PPO activity increased by about 10 and 15% under GMG+M and GMG treatments, respectively, when compared to the control. The two treatments were significantly different from each other, with GMG proving higher PPO activity values than GMG+M (Figure 5).

## 3. Discussion

### 3.1. Supply of Glucomoringin and Moringin

In recent years, secondary metabolites have gained increasing interest as phytochemical candidates for potential use as natural products in crop protection [38], also allowing for the valorization of agro-industrial waste in a circular economy perspective [39]. Particularly, the presence of glucosinolates has been linked to the multifunctional properties of Brassicaceae vegetable wastes and byproducts, including antifungal and antibacterial potential [40]. Glucosinolates, 159 chemical structures known to date, occur throughout the order Brassicales along with the presence of specific thioglucosidase enzymes, the myrosinases, which can hydrolyze these compounds to a variety of degradation products [41]. The glucosinolate–myrosinase system is recognized as a part of the plant’s endogenous chemical defense against susceptible pathogens and pests [42]. Abdel-Farid et al. (2010) [43] found that infection with *F. oxysporum* triggers the accumulation of glucosinolates in *Brassica rapa* in a manner directly related to the resistance of the cultivars panel tested. In plants, myrosinase is located in specialized cells known as myrosin cells/idioblast cells of the phloem parenchyma, whereas the glucosinolates are held separately in the vacuoles or S-cells of most tissues. The activation of the glucosinolate–myrosinase system due to the breakdown of the cell compartmentalization by pathogens, leading to the formation of isothiocyanates, is the most accepted mode of defensive action [44].

Moringa seed cake, the byproduct of oil extraction from *M. oleifera* seed, is a rich source of biologically active molecules that may be used as promising sustainable alternatives for many applications, providing added value to what may be regarded as a side-product [45]. In addition to the flocculant protein useful for water treatment, moringa seed cake is a valuable source of GMG, an atypical member of the glucosinolate family, precursor of the isothiocyanate GMG+M. Notably, moringa seed cake meets the criteria for a convenient purification of GMG on the multigram scale. Indeed, the raw material used in the presented research contained GMG as a single glucosinolate in a remarkably high percentage (13.9% *w*/*w*), according to the level range already reported for the whole seed (8–10% *w*/*w*) [23]. Otherwise, GMG is commercially available only as an analytical standard at a relatively high cost. The availability of GMG purified at our lab prompted us to test it, either as an intact glucosinolate and after preincubation with myrosinase, as a potential biopesticide for controlling FOL in tomato. The strategy of the in situ biotransformation of GMG enabled us to obtain GMG+M as a fresh, ready-to-use solution, avoiding the need for any further chromatographic purification step to isolate the pure compound. Furthermore, Lu et al. (2021) [46] observed that although GMG+M generally shows a gradual degradation with increasing temperature according to pseudo-first-order reaction kinetics, its amount in aqueous suspension remained constant over a period of one hour up to 50 °C. The labile nature of GMG+M is a critical issue that suggests the need to focus on stability and formulation [23] to extend the product shelf life and the technology translational potential.

### 3.2. Antifungal Properties and Seed Coating Efficacy

In this study, both in vitro spore germination and mycelial development of FOL were significantly inhibited following exposure to GMG+M solutions applied in the range 1–1000 µM, showing a dose-dependent response. Conversely, treatments with the precursor GMG did not differ significantly from the non-treated control. The results of in vitro tests indicated that the direct antifungal effects of purified GMG are related to its biotransformation mediated by myrosinase into the corresponding isothiocyanate GMG+M.

Naturally occurring isothiocyanates derived from plant glucosinolates are electrophilic compounds due to the high reactivity of the electron poor carbon of the isothiocyanate group [47,48] displaying antifungal properties, which are associated with multitarget mechanisms causing degradation of proteins, peptides, and/or amino acids and a redox imbalance through interference with glutathione homeostasis, leading to cell membrane rupture, hyphal deformity, and loss of cellular electrolytes [49]. In particular, GMG+M has shown a wide array of biological activities, including antimicrobial properties [22], through molecular mechanisms affecting cell integrity and microorganism morphology [50]. Exposure of *Listeria monocytogenes* [51] and *Staphylococcus aureus* [52] to GMG+M resulted in severe impairment of cell membrane integrity with macromolecule leakage, induction of oxidative stress and threat to bacterial viability. On the other hand, to the best of our knowledge, this is the first study to test standardized solutions of GMG+M for its antifungal activity since there are no other studies on its impact on the selected fungal target FOL using the pure compound. An earlier study, Eilert et al. (1981) [53], reported the antifungal activity of an aqueous extract of *M. oleifera* seeds tested in the Petri plate diffusion assay against several fungi. The extract was moderately effective against FOL and more active against other phytopathogens, including *Botrytis allii*, *Phytophthora cactorum*, and *Piricularia oryzae* [53]. Several studies, instead, focused on the effects of commercially available isothiocyanates, i.e., allyl isothiocyanate (3-isothiocyanatoprop-1-ene) on *Fusarium solani* [54], ethyl, butyl, phenylethyl, and benzyl isothiocyanate against *F. oxysporum* pathogenic isolates of conifer seedlings [55], also affecting cell wall and membrane integrity.

In accordance with in vitro results, preventive treatment by coating tomato seeds with GMG+M (100 µM) resulted in an effective reduction (ca 70%) of post-emergence wilt severity caused by FOL. The antifungal effectiveness, combined with a good stability of the freshly prepared water solution, prompted us to explore the use of GMG and GMG+M for in vivo seed prophylaxis, as evaluated in the current study, although with the limitation of testing only one concentration (100 µM) of the two phytochemicals and the lack of a direct comparison with a commercial synthetic fungicide. The levels of disease control observed here are comparable to the reduction achieved in recent studies on the same pathosystem by biocontrol agents belonging to the genera *Trichoderma* [56] and *Peribacillus* [57]. The in vivo disease assay provided proof-of-concept for seed coating efficacy; however, the use of a single concentration represents a drawback that needs to be addressed in future industrial development trials by comparing the proposed phytochemical treatments with a standard conventional fungicide to benchmark their scalability and evaluate long-term effects on yield levels.

In vivo experiments showed that the intact precursor GMG was able to produce the same control effect as its biotransformed product. Interestingly, in contrast to the in vitro response in the Petri plate, the ability to control the development of the disease in tomato seedlings was statistically comparable between the two treatments. Considering our in planta experimental conditions that let us exclude any plausible involvement of extracellular myrosinase in quick activation of hydrolytic reactions, it is reasonable to assume that the control effect is based on a plant-mediated response to the intact glucosinolate GMG treatment. As a matter of fact, studies on the topic have shown that the exogenous application of glucosinolates can also lead to physiological benefits in plants. Spraying the tomato foliage with glucosinolate-based formulations, for example, improved yields by protecting the plants and promoting harmonious vegetative growth and health [58]. The treatment of broccoli seeds with extracts rich in glucosinolates and phenols stimulated primary and secondary metabolism, resulting in improved seedling development, increased biomass yield, detoxification of reactive oxygen species, decreased lipid peroxidation, and a substantial increase in phytohormones [59]. The hypothesis of a plausible mechanism involving plant-mediated action is supported by the fact that both GMG and GMG+M seed coatings triggered polyphenol oxidase (PPO) activity in five-day-old tomato sprouted rootlets. PPO is an enzyme that is activated following the cleavage of its C-terminus by a protease in response to stress or environmental changes, and its upregulation has been linked to resistance response to various pathogens in tomato [60], including FOL [61]. However, the mechanistic studies are merely preliminary, as only PPO activity was measured. Future work should include additional defense-related enzymes (PAL, POD, CAT) and transcriptomic analysis to fully characterize the plant-mediated response.

### 3.3. Literature Brief Review on Mechanistic Hypothesis

Based on current knowledge, glucosinolates exert their antifungal action essentially through their hydrolysis products, while direct molecule-fungus mechanisms have not been previously reported [62]. The conversion of these secondary metabolites into the corresponding isothiocyanates can be catalyzed by both intra- and extracellular enzyme systems. Actually, myrosinase-like enzymes could be carried by a lot of microorganisms, including components of the intestinal microbiota or those present in soil or food matrices. This has been recently reported in a systematic review detailing the conversion mechanisms of glucoraphanin—the renowned dietary glucosinolate found in broccoli and Tuscan black kale—in both plants and microorganisms [63]. In this regard, the myrosinase activity in the soil influences the shelf life of glucosinolates, as it is positively correlated with their degradation kinetics, as is evident in applications involving biofumigation [64,65].

Given the preliminary nature of the mechanistic data emerging from this study, further experiments are needed to confirm the hypothesis that GMG may promote a plant-mediated mechanism. A comprehensive review of the literature sheds light on the putative role of glucosinolates as signaling molecules.

Glucosinolates together with reactive oxygen species were found to be involved in multiple signaling pathways modulating the permeability of plasmodesmata, openings that allow direct communication between cells across the cell wall [66]. Plasmodesmata exclusion selectivity modulates the cell-to-cell movement of FOL effector proteins necessary for full virulence [67]. The accumulation of callose, a *β*-1,3-glucan polysaccharide, in the cell wall surrounding plasmodesmata to limit molecular traffic between neighboring cells is considered part of the plant’s early defense response to the pathogen [68,69,70]. Interestingly, exogenous application of glucosinolates, but not their degradation products, on *Arabidopsis thaliana* was found effective in regulating plasmodesmata size, just via callose deposition, suggesting a putative role in interfering with *Fusarium* effectors’ intercellular traffic [71]. As a matter of fact, the formation of new callose barriers with plasmodesma obstruction has been proposed as a strategy to stop the advance of the pathogen, as observed at the ultrastructural level in the incompatible reaction (resistant plant) carnation/*Fusarium oxysporum* f. sp. *dianthi* compared to the compatible one (susceptible plant) [72].

Finally, the findings of PPO activity could also be consistent with the central theme of the plant-mediated GMG action hypothesis under consideration. Indeed, PPO hydroxylates monophenol to o-diphenol, and it oxidizes o-diphenol into o-quinone. Furthermore, o-quinone may be involved in resistance to biotic stress through polymerization and condensation with amino acids and proteins to produce brown substances or with other phenols to produce melanin as a physical barrier, or through its redox reaction that generates ROS, causing oxidative burst [73].

## 4. Materials and Methods

### 4.1. Isolation and Purification of Glucomoringin

GMG was obtained from *M. oleifera* seed cake (PKM-2 cake powder; Indena India Pvt Ltd., Bangalore, India) according to a previously reported method, with some modification. Briefly, the cake powder (100 g) was treated with boiling water (1 L) in order to quickly deactivate the endogenous myrosinase enzyme. GMG was extracted using a VDI 25 homogenizer (VWR, Darmstadt, Germany) and then centrifuged with a 5810R centrifuge (Eppendorf AG, Hamburg, Germany) at 4000 rpm for 10 min at 10 °C. The supernatant was then purified in two sequential chromatographic steps, according to a well-established procedure available at our lab and already reported extensively in a previous study [35]. GMG was first isolated by anion exchange chromatography, loading the water extract by gravity on an open Econo-Column (25 × 200 mm, Bio-Rad Laboratories, Inc., Hercules, CA, USA) filled with DEAE-Sephadex A-25 resin (GE Healthcare, Milan, Italy) conditioned with acetate buffer (25 mM, pH 5.6). After washing with distilled water (1 L), GMG was eluted with a water solution of K_2_SO_4_ (0.2 M, 500 mL). The eluate was dried using a rotary evaporator (model RV3, IKA-Werke GmbH & Co. KG, Staufen, Germany) at 60 °C under reduced pressure. Boiling methanol (3 × 100 mL) was then added to the solid to extract GMG. After removing the remaining particles of K_2_SO_4_ by filtration, the methanolic extract was dried by rotatory evaporation. The purity of GMG was further improved by gel-filtration using an XK 26/100 column packed with Sephadex G10 (GE Healthcare, Milan, Italy) connected to a fast protein liquid chromatograph system (ÄKTA™ FPLC System, GE Healthcare, Milan, Italy). The isolated GMG powder was dissolved in ultrapure water (0.5 g mL^−1^), filtered through a 0.45 μm membrane filter (Gema Medical S.L., Barcelona, Spain), and charged (2 mL) onto the column. HPLC-grade water was used as the mobile phase at a flow rate of 2.0 mL min^−1^, and the eluate absorbance was monitored at 254 nm. After the void volume was discarded, collected fractions (6 mL) were analyzed by HPLC-PDA and those containing GMG as a single glucosinolate were pooled and freeze-dried, yielding a fine white powder with purity determined as described in Section 4.2.

### 4.2. Glucomoringin Extraction, Desulfation and HPLC Analysis

The content of GMG was assessed over the entire purification workflow, starting with the raw material and ending with the final purified compound, by high-performance liquid chromatography (HPLC) analysis of the desulfo-derivative, according to the EU standard procedure ISO 9167:2019 [74].

For quantification of GMG in the raw material, duplicate PKM-2 powder samples (200 mg) were extracted for 5 min at 80 °C twice with ethanol/water (5 mL, 7:3 *v*/*v*), using a VDI 25 homogenizer (VWR, Darmstadt, Germany) and then centrifuged with a 5810R centrifuge (Eppendorf AG, Hamburg, Germany) at 4000 rpm for 10 min at 10 °C. Supernatants were combined and then treated as follows.

PKM-2 hydroalcoholic extracts (1 mL), PKM-2 water extracts (1 mL), chromatography fractions (1 mL), as well as water solutions of purified GMG (1 mL), were loaded onto a mini-column filled with DEAE-Sephadex A-25 anion-exchange resin (0.6 mL, GE Healthcare, Milan, Italy) conditioned with 25 mM sodium acetate buffer (pH 5.6). After washing with buffer (3 mL), sulfatase purified by fractional precipitation (200 μL) [75] was loaded onto the mini-column and left for overnight reaction. Desulfation was performed in triplicate. Desulfo-glucosinolates were then eluted with HPLC-grade H_2_O (3 mL) and analyzed (injection volume: 20 µL) on an HPLC (Shimadzu, Kyoto, Japan) system equipped with an Avantor ACE 5 C18 column (250 × 4.6 mm, 4 μm particle size) (VWR, Darmstadt, Germany) thermostated at 30 °C and having a PDA detector. The chromatography was performed at a flow rate of 1 mL min^−1^, eluting with a gradient of H_2_O (A) and CH_3_CN (B) following the program: 1 min 1% B; 25 min linear gradient up to 25% B; 3 min linear gradient down to 1% B. Desulfo-glucosinolates were detected by absorbance monitoring at 229 nm. Identification of desulfo-glucomoringin (ds-GMG) peak was performed based on retention time and UV spectra of a purified standard available at our lab. GMG content was quantified by using a calibration curve of pure DS-sinigrin solution (range from 0.06 to 1.93 mM) obtained from commercial sinigrin (prop-2-enyl glucosinolate analytical standard, Sigma Aldrich, St. Louis, MO, USA).

### 4.3. Myrosinase-Catalyzed Hydrolysis of Glucomoringin

GMG+M solutions for in vitro and in planta studies were freshly prepared through GMG transformation with commercial myrosinase enzyme from *Sinapis alba* (white mustard) seed (Sigma-Aldrich, St. Louis, MO, USA). Purified GMG powder (6.1 mg/mL) was dissolved in phosphate-buffered solution pH 7.2 and hydrolyzed with myrosinase (3 U/mL, 100 µL) at 37 °C for 1 h, yielding GMG+M quantitatively, as previously reported [35,36,37]. One myrosinase unit was defined as the amount of enzyme able to hydrolyze 1 μmol min^−1^ of sinigrin at neutral pH and 37 °C. Hydrolysis of GMG was monitored by HPLC-PDA until quantitative conversion to GMG+M. Mixture samples were directly analyzed on an HPLC (Shimadzu, Kyoto, Japan) system equipped with a Synergi Fusion-RP column (250 × 4.6 mm, 4 μm particle size) (Phenomenex, Torrance, CA, USA), thermostated at 30 °C, and having a PDA detector. The chromatography was performed at a flow rate of 1 mL min^−1^, eluting with a gradient of H_2_O (A) and CH_3_CN (B) following the program: 1 min 1% B; 33 min linear gradient up to 33% B; hold at 33% B for 10 min; 3 min linear gradient down to 1% B. Elution of GMG and GMG+M was detected by monitoring the absorbance at 229 nm.

### 4.4. Plant Pathogen

The fungal pathogen used in this study was *Fusarium oxysporum* f. sp. *lycopersici* strain ATCC 16605, the causal agent of tomato wilting, which was maintained in the collection of CREA Research Centre for Vegetable and Ornamental Crops (Pontecagnano Faiano, Italy) at −80 °C on potato dextrose broth (PDB, Condalab, Madrid, Spain) amended with 20% (*v*/*v*) glycerol. The pathogen was cultured for current uses on potato dextrose agar (PDA, VWR International BVBA/SPRL, Leuven, Belgium) in 90 mm-Petri plates and incubated at 25 °C in the darkness. Conidia of the fungus were collected in water suspension by flooding 10-day-old PDA cultures with sterile distilled water that was gently spatulated with a sterile bent plastic rod to release conidia, recovered with a 3 mL plastic pipette, filtered through synthetic filtering wool, and kept at the desired concentration by using a Burker chamber (Paul Marienfeld GmbH & Co. KG, Lauda-Königshofen, Germany) for enumeration.

### 4.5. Evaluation of the In Vitro Antifungal Activity

Antifungal effects of the phytochemical preparations were assessed in liquid microcultures on (a) conidia germination and (b) mycelial growth. Filter-sterilized (0.22 μm filter pore size) GMG and GMG+M solutions were poured into 0.1 × Czapek Dox Broth (Liofilchem, Roseto degli Abruzzi, Italy) to be assayed at four final concentrations: 1, 10, 100 and 1000 µM; while non-amended broth was used as reference control.

The conidia germination assay was carried out in 0.5 mL microtubes, while the mycelial growth assay was carried out in 96-well microplates. Each microtube or well was poured with 100 μL of the broth and inoculated with the spore suspension to reach the final concentration of 1 × 10^5^ conidia mL^−1^. Treatments were incubated at 25 °C in the dark for four days, and the percentage of conidia germination rate (CGR%) was assessed daily by observing at least 100 spores per replicate under a light microscope and expressed as follows [76]

$$\mathrm{Conidia}\mathrm{Germination}\mathrm{Rate}\%=\frac{N{}^{\circ}\mathrm{of}\mathrm{germinated}\mathrm{conidia}\mathrm{on}100\mathrm{of}\mathrm{the}\mathrm{treatment}}{N{}^{\circ}\mathrm{of}\mathrm{germinated}\mathrm{conidia}\mathrm{on}100\mathrm{of}\mathrm{the}\mathrm{control}}\times 100$$

Mycelial growth rate in each well was assessed daily for four days–24, 48, 72, 96 h post-inoculum–spectrophotometrically by determining the light dispersion in the media through absorbance readings at 595 nm wavelength using the Bio-Rad Microplate Reader 550 (Bio-Rad, USA). The experimental design included 3 replicates (3 wells) for each treatment (phytochemical solution × concentration), and the experiments were performed twice.

### 4.6. Evaluation of the In Vivo Disease Control Ability on Tomato Plants

Tomato seeds (*S. lycopersicum* cv Crovarese, Semiorto Sementi, Sarno, Italy) were surface sterilized by immersion in 1% hypochlorite for 2 min, then in 70% ethanol for 2 min, and finally rinsed three times in distilled water. The seeds were coated by pipetting 5 µL per seed of 100 µM GMG or GMG+M solution onto their coat and then allowed to dry on parafilm under laminar airflow for 8 h at room temperature. Seeds coated only with water were used as a control reference. Afterwards, testing seeds were sown (10 per pot, obtaining on average 8 plants in each) at 0.5 cm depth, in 12 cm diameter plastic pots (5 replicates per experiment) containing pre-autoclaved soil-peat inoculated with 2% (*w*/*w*) FOL-infected millet. This inoculum was previously prepared by infesting autoclaved 0.1 × PDB-saturated millet seeds with pieces of FOL PDA culture (10 plugs per 100 g), left to incubate in the dark at 28 °C for 21 days, and homogenizing the biomass in a mortar before pouring pots. Non-infected soil peat was used as growing media for healthy control plants. Pots were placed in a completely randomized design in a climatic chamber at 25 °C with a 12 h photoperiod. After 21-day incubation, wilting magnitude was assessed using the scoring 0–3 (0 = plant without symptoms; 1 = growth stunting; 2 = plants with yellow leaves and reduced growth; maximal score of 3 = wilting, necrosis, or dead plants) [77] and expressed as disease incidence percentage (DI %), as follows

$$\mathrm{DI}\%=\frac{\sum \mathrm{plants}\mathrm{with}\mathrm{symptoms}}{\mathrm{total}\mathrm{number}\mathrm{of}\mathrm{plants}}\times 100$$

and as Mc Kinney disease severity percentage (DS %) [78] according to the following formula:

$$\mathrm{DS}\%=\frac{\sum \mathrm{class}\mathrm{frequency}\times \mathrm{class}\mathrm{score}}{\mathrm{total}\mathrm{number}\mathrm{of}\mathrm{plants}\times \mathrm{maximal}\mathrm{score}}\times 100$$

The in vivo experiment was carried out twice.

### 4.7. Polyphenol Oxidase Activity Assay in Tomato Sprouted Rootlets

Plant stress oxidative enzyme activity was detected to evaluate plant response to GMG and GMG+M seed coating. Before coating, tomato seeds cv. Crovarese (La Semiorto Sementi srl, Sarno, Italy) was sterilized two times using 1 % hypochlorite for 2 min and 70 % ethanol for 2 min. Seeds were then washed three times with distilled water. Afterwards, tomato seeds were dipped into 100 µM GMG and GMG+M solution (4 μL for seed supplemented with 0.05 % *v*/*v* Tween-20) on parafilm and were left to dry in laminar airflow at room temperature until water was thoroughly evaporated. Tomato control seeds were treated only with distilled and sterile water. Coated tomato seeds were placed in 90 mm Petri dishes containing bibulous paper supplemented with 2 mL of sterile and distilled water at room temperature and kept in the dark for germination. Polyphenol oxidase (PPO) activity was assessed on plant tissue samples of five-day-old sprouted rootlets (20 mg). The assay was carried out using the PPO Activity Assay Kit (Elabscience^®^ Texas, Houston, TX, USA), according to the manufacturer’s protocol. PPO activity was expressed as a ∆OD value at 410 nm per min at 37 °C.

### 4.8. Statistics

From the in vitro experiments, data of FOL mycelial growth (abs. at 595 nm values) and those of conidia germination rate (CGR%) after transformation in angular value (arcsine square root), were subjected to repeated measures analysis of variance (RM-ANOVA). Since no significant experimental effect was found, data from repeated experiments were pooled. Treatments (Tr, GMG and GMG+M) and concentrations (C, in the range 0–1000 µM) were considered as fixed effects, as well as their interaction Tr × C; while the Time (Ti) was considered to have a random effect—as well as the interactions Ti × Tr, Ti × C, and Ti × Tr × C. From the in planta experiments, disease severity % (DS%) data were first transformed into an angular value and then, after exclusion of the experiment factor due to the absence of significant effects, were subjected to one-way ANOVA. All the means were separated by Tukey’s honest significant difference (HSD) test for *p* ≤ 0.05.

## 5. Conclusions

This study highlighted the potential biotechnological value of *M. oleifera* seed cake for the development of a biopesticide. To our knowledge, this is the first investigation of the in vitro effect of pure glucomoringin and standardized moringin solutions on the conidia germination and mycelial growth of *Fusarium oxysporum* f. sp. *lycopersici*. Importantly, in vivo disease assays proved both phytochemicals as promising seed coating agents in reducing vascular wilt in tomato plants. Some mechanistic hypotheses have been derived by comparing the experimental observations from the current study with data from the literature, suggesting that further research is needed to clarify how glucomoringin application may play a role in complex plant–pathogen interactions. Future research could therefore focus on analyzing direct evidence of the mechanisms of action, optimizing concentration values application, and developing formulations, all with a view to transferring this innovation to real-world conditions in field trials. Moreover, glucomoringin and moringin formulation tests will be necessary to enhance the seed coating capacity and to extend the shelf life of coated seeds for the seed market.

## Figures and Tables

**Table 1:** Synthetic results of repeated measures analysis of variance (RM-ANOVA) on *Fusarium oxysporum* f. sp. *lycopersici* (FOL) mycelial growth and conidia germination data. SS: sum of squares; DF: degree of freedom; MS: mean squares; F: F-statistic; *p*: *p*-value.

	FOL Mycelial Growth (Abs. 595 nm)					FOL Conidia Germination Rate (%)				
Source of Variance	SS	DF	MS	F	*p*	SS	DF	MS	F	*p*
Tr ^1^	6,502,688	1	6,502,688	4449.04	<0.001	17.05833	1	17.05833	633.087	<0.001
C ^2^	1,249,853	4	312,463	213.78	<0.001	0.57341	4	0.14335	5.320	<0.001
Tr × C	1,498,205	4	374,551	256.26	<0.001	4.27045	4	1.06761	39.622	<0.001
Error	73,080	50	1462			1.34723	50	0.02694		
Ti ^3^	2,497,765	3	832,588	1723.96	<0.001	4.65317	3	1.55106	568.768	<0.001
Ti × Tr	773,086	3	257,695	533.58	<0.001	0.55446	3	0.18482	67.772	<0.001
Ti × C	150,973	12	12,581	26.05	<0.001	2.77103	12	0.23092	84.677	<0.001
Ti × Tr × C	156,012	12	13,001	26.92	<0.001	0.24309	12	0.02026	7.428	<0.001
Error	72,443	150	483			0.40906	150	0.00273		

Tr ^1^: treatment, C ^2^: concentration, Ti ^3^: time.

## Data Availability

The original contributions presented in this study are included in the article. Further inquiries can be directed to the corresponding author.
